# Integrating Primary Health Care into Flexible Surge Capacity: Bridging the Subnational “Missing Middle” in Health Emergency Preparedness

**DOI:** 10.3390/healthcare14183074

**Published:** 2026-09-18

**Authors:** Amir Khorram-Manesh

**Affiliations:** 1 Department of Surgery, Institute of Clinical Sciences, Sahlgrenska Academy, Gothenburg University, 41345 Gothenburg, Sweden; amir.khorram-manesh@surgery.gu.se; Tel.: +46-(0)-707-72-27-41; 2 Center for Disaster Medicine, University of Gothenburg, 40530 Gothenburg, Sweden; 3 Gothenburg Emergency Medicine Research Group (GEMREG), Sahlgrenska University Hospital, 41345 Gothenburg, Sweden

**Keywords:** primary health care, disaster preparedness, emergency management, flexible surge capacity, health system resilience, community resilience, surge capacity, subnational health systems

## Abstract

**Highlights:**

**What are the main findings?**

- Primary health care (PHC) performs essential emergency and continuity functions, but these functions have not been explicitly integrated into the surge capacity/flexible surge capacity (FSC) concept at the subnational level.
- This review integrates two complementary bodies of literature—emergency-ready PHC and FSC—to identify areas of convergence, required adaptations, safeguards, and testable hypotheses for PHC-integrated FSC.

**What are the implications of the main findings?**

- PHC could be explicitly incorporated into subnational FSC preparedness, including governance, capability mapping, continuity planning, exercises, activation criteria, and protection of routine essential services.
- Flexible surge capacity should be developed before emergencies occur, with clear responsibilities, funding, training, activation criteria, safeguards, and evaluation measures that are adapted to local health system and community contexts.

**Abstract:**

Health emergencies are governed nationally but experienced locally, where primary health care (PHC) plays a central role in maintaining essential services, surveillance, medication access, chronic disease management, communication, and recovery. Flexible surge capacity (FSC) was developed as a cross-sector approach for identifying and preparing additional personnel, facilities, supplies, systems, and community capabilities, but PHC has not been explicitly integrated into the concept. This narrative conceptual review examined how emergency-ready PHC can be incorporated into FSC at the subnational level. The literature identification followed two complementary streams: one addressing PHC and health-emergency preparedness and another concept-directed stream addressing the foundational and subsequent FSC literature. Following relevance screening, 31 core publications were retained for conceptual synthesis. The two evidence streams were compared to identify functional convergence, required adaptations, governance and clinical safeguards, activation considerations, and evidence gaps. The resulting framework positions PHC as defining essential functions that should be sustained during emergencies, while FSC provides additional cross-sector capability when routine functional capacity becomes insufficient. The proposed PHC-integrated FSC model therefore represents a continuity-and-surge approach rather than solely an expansion of acute-care capacity. The model is conceptual, and its downstream effects should be regarded as hypotheses for future evaluation rather than established effectiveness claims. PHC-integrated FSC should complement, not replace, adequately resourced routine PHC and existing emergency-response systems.

## 1. Introduction

Health emergencies are planned through national policies and emergency management structures but are experienced in local communities [1]. Pandemics, earthquakes, floods, cyclones, wildfires, conflict, cyber disruption, and infrastructure failure create acute demand while simultaneously interrupting routine care [2,3]. Hospitals are central to severe illness and injury, but much of the population continues to depend on primary health care (PHC) for first-contact assessment, medicines, chronic disease management, prevention, vaccination, mental health support, and trusted information. The long-standing PHC and disaster literature therefore argues that effective emergency preparedness cannot be confined to hospitals and specialist emergency services [2,3,4]. The World Health Organization (WHO) has similarly emphasized PHC as a foundation for resilient health systems and for maintaining essential services during emergencies [4,5].

The WHO Emergency Ready Primary Health Care Framework, released in 2026 with an associated operational guide, organizes readiness around four interdependent capacities: connect, detect, protect, and treat [6,7]. These capacities position PHC within communication, surveillance, risk recognition, workforce and community protection, clinical management, referral, and continuity of essential services. Their value, however, depends on translation into arrangements that function across geographical and organizational settings. National frameworks establish direction, but cities, provinces, regions, districts, municipalities, and local health networks must connect the organizations and communities that deliver care [6,8].

Recent discussion in Aotearoa New Zealand illustrates this wider international challenge. PHC contributed to identification, testing, vaccination, continuity of care, and community recovery during COVID-19, the Canterbury earthquake sequence, and climate-related emergencies, yet its formal role in emergency planning and response remains incompletely defined [9,10]. Gray and Murton recently called for stronger connections between PHC, health authorities, emergency management, and communities, highlighting a continuing tendency toward hospital-centered preparedness [9]. Similar concerns have been reported internationally, where primary-care professionals may be relied upon during crises despite limited involvement in planning, role definition, training, and exercises [2,11].

These discussions also reveal an important terminological issue. The terms *primary health care*, *primary care*, and *community services* are sometimes used interchangeably, although they refer to different levels of function and organization. In this review, PHC is used in the broader WHO sense to describe a health system approach encompassing first-contact care, prevention, population health, community engagement, and coordination [4,8]. *Primary care* is used more narrowly to denote frontline clinical services delivered by general practitioners, family physicians, nurses, and associated professionals [12]. Community services and community organizations are considered collaborative actors within the wider PHC environment but are not treated as synonymous with PHC. This distinction is important because the proposed integration with surge capacity concerns both clinical primary-care functions and the broader coordinating and community-facing functions of PHC.

Against this background, two related but incompletely connected bodies of literature can be identified. The first concerns emergency-ready PHC: the functions that PHC must maintain during crises, its contribution to detection, protection, treatment, communication, referral, and continuity of essential services, and its coordination with hospitals, public health, emergency management, and communities [2,3,4,5,6,7,8,9,10,11].

The second concerns flexible surge capacity (FSC), which was developed as an approach for pre-identifying and preparing additional personnel, facilities, supplies, logistics, systems, and community capabilities that can be mobilized across organizational boundaries when routine functional capacity becomes insufficient [13,14]. FSC originated within the present research program and has subsequently been examined through conceptual work, feasibility studies, exercises, and context-specific applications [13,14,15,16,17]. However, PHC has not been explicitly developed as a central component of the FSC concept.

The unresolved question is therefore not whether a subnational governance level exists, but how emergency-ready PHC can be incorporated into FSC at the operational interface between national preparedness and frontline delivery. The aim of this narrative conceptual review was to integrate the PHC/emergency preparedness and FSC studies to identify: (1) PHC functions and continuity requirements relevant to surge planning; (2) FSC capabilities that may support those functions; (3) adaptations and safeguards required when PHC is incorporated into FSC; and (4) evidence gaps requiring future empirical evaluation. The resulting framework conceptualizes the subnational “missing middle” as an operational coordination interface rather than as a new administrative tier.

## 2. Materials and Methods

A narrative conceptual review was undertaken because the aim was to integrate two heterogeneous but complementary bodies of literature and to develop a conceptual model rather than estimate intervention effects [18,19,20]. The review was structured a priori around two evidence streams: (1) primary health care (PHC) and health-emergency preparedness, used to identify PHC emergency functions, continuity requirements, organizational challenges, and implementation safeguards; and (2) flexible surge capacity (FSC), treated as the pre-existing surge concept being examined for possible integration with PHC.

### 2.1. Literature Identification

Initial exploratory searches using broader combinations of PHC, emergency preparedness, disaster preparedness, surge capacity, continuity of essential services, health system resilience, community participation, and subnational health systems produced many records but did not reliably identify the specific literature on flexible surge capacity. Because FSC was introduced in 2020 and the purpose of this review was to examine how PHC could be incorporated into that pre-existing concept, the formal database search window was limited to publications from 2020 through July 2026 (Supplementary Table S1).

For the PHC/emergency-preparedness stream, PubMed, Scopus, and Web of Science were searched using the same final search string: “primary health care” AND “primary care” AND “health emergency preparedness.” Searches were conducted between 1 and 15 July 2026 and were limited to English-language publications published from 2020 through July 2026. The database searches yielded 109 records: 19 from PubMed, 70 from Scopus, and 20 from Web of Science.

For the FSC stream, PubMed, Scopus, and Web of Science were searched using the exact term “flexible surge capacity.” Known foundational FSC publications were supplemented through backward reference-list screening and forward citation tracking. Although the formal database window began in 2020, earlier publications were retained when they directly informed concepts necessary to interpret PHC, surge capacity, preparedness, coordination, or related implementation mechanisms. The database searches between 1 and 15 July 2026 yielded 132 records: 20 from PubMed, 108 from Scopus, and 4 from Web of Science.

The two database streams therefore generated 241 records before removal of duplicates and relevance screening. Because this was a narrative conceptual review and duplicate and exclusion counts were not prospectively recorded at each screening stage, the search process is presented as a descriptive search-and-synthesis flow rather than as a PRISMA-compliant systematic review flow diagram.

The literature from Aotearoa New Zealand was retained, where relevant, as an illustrative contemporary example of PHC participation in emergencies and of the challenge of connecting PHC with health authorities, emergency management, and communities; it was not treated as the geographical focus of the review [9,10].

### 2.2. Eligibility and Selection

The formal database searches focused on publications from 2020 onward because FSC was introduced in 2020. Earlier publications identified through reference screening or already established as foundational to PHC, surge capacity, disaster preparedness, or related concepts were retained when directly relevant to the conceptual synthesis. Sources were screened and selected by the author. Because this was a single-author narrative conceptual review, no inter-reviewer disagreement procedure was applicable.

Sources were retained when they contributed substantially to at least one of four analytical domains:

1. PHC emergency functions and continuity requirements;
2. subnational or multisectoral coordination between national preparedness and frontline delivery;
3. defining features, implementation experience, or limitations of FSC; or
4. governance, workforce, information, clinical safety, community participation, equity, or activation issues relevant to integrating PHC into FSC.

**Figure 1:**
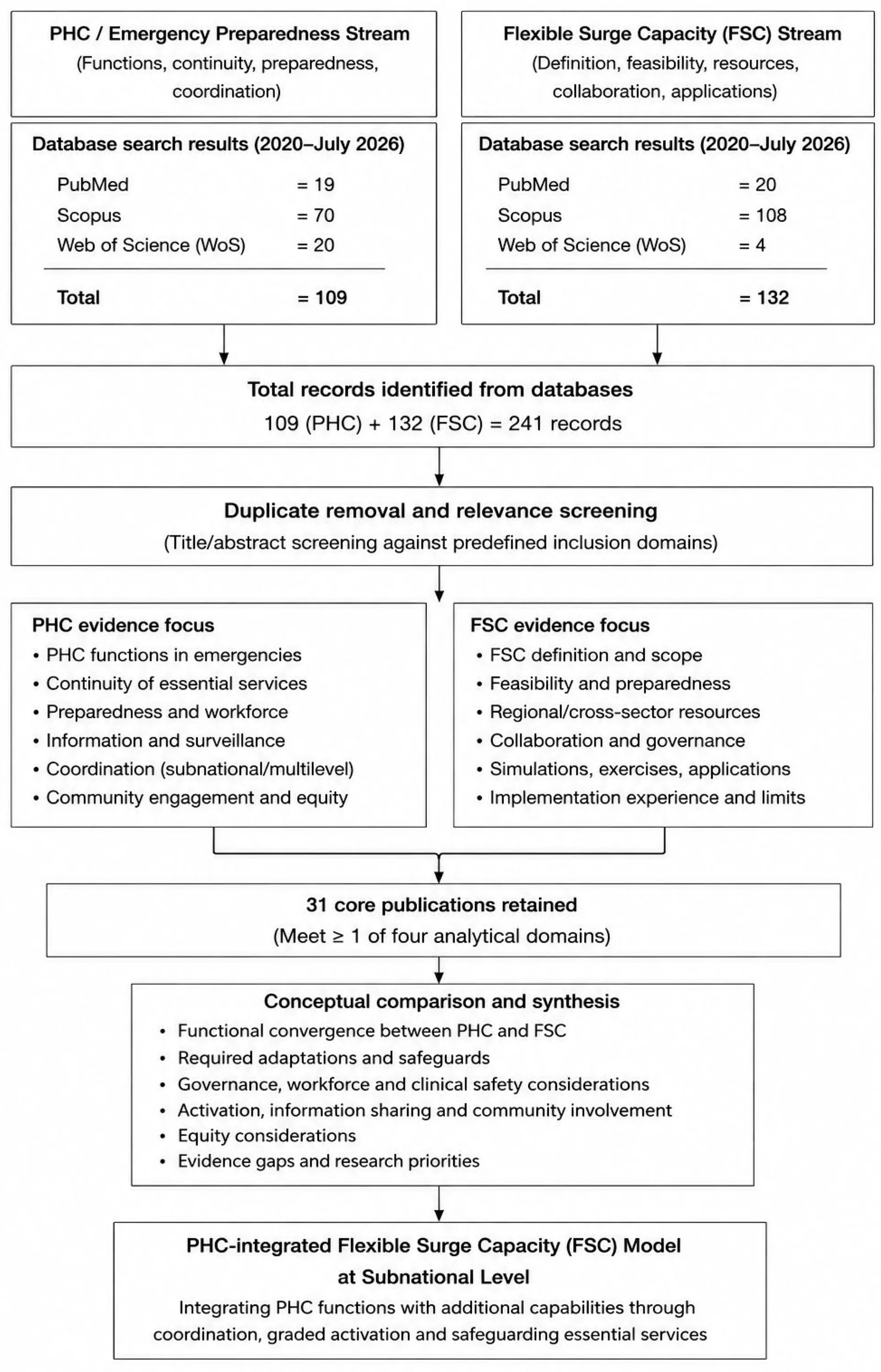
Search and conceptual synthesis process. Two complementary evidence streams were examined: PHC and health emergency preparedness, and the concept-directed FSC literature. Database searches identified 109 PHC-related records (PubMed 19, Scopus 70, Web of Science 20) and 132 FSC-related records (PubMed 20, Scopus 108, Web of Science 4), giving 241 records before duplicate removal and relevance screening. Following screening against the predefined analytical domains, 31 core publications were retained for conceptual synthesis. The two evidence streams were then compared to identify areas of functional convergence, required adaptations and safeguards, activation considerations, and remaining evidence gaps, which informed the proposed PHC-integrated FSC model. The figure presents a descriptive narrative-review process and is not intended as a PRISMA-compliant flow diagram.

Sources focused exclusively on unrelated hospital surges, non-health emergency management, or topics without a direct connection to these domains were excluded. Following relevance screening and removal of duplicate or tangential records, 31 publications were retained as the core evidence base for the conceptual synthesis (Supplementary Table S2 and Figure 1).

To reduce the subjectivity inherent in single-author selection, eligibility was guided by predefined analytical domains, with the two evidence streams assessed separately before conceptual synthesis.

### 2.3. Conceptual Synthesis

The two streams of evidence were initially examined separately. The PHC stream was used to identify the functions that should be maintained or strengthened during emergencies and the organizational conditions affecting their delivery. The FSC stream was used to identify the resources, collaborative mechanisms, implementation experience, and limitations of the existing FSC concept.

The two streams were subsequently compared to determine: (1) where PHC functions corresponded to FSC capabilities; (2) where incorporating PHC would require adaptation of FSC; (3) which governance, workforce, clinical, information, community, equity, and activation safeguards would be required; and (4) which proposed relationships remained unsupported by direct empirical evidence. Recurring concepts were organized into a framework that linked emergency-ready PHC functions with FSC capabilities, subnational coordination, graded activation, and enabling conditions.

No formal risk-of-bias assessment or quantitative synthesis was undertaken because the review was narrative and conceptual rather than systematic [18,19,20,21]. The purposive FSC stream is not presented as a comprehensive review of all the surge capacity literature; its purpose was to represent the literature defining and developing the specific FSC concept being considered for integration with PHC. The implications of this approach for selection bias and intellectual allegiance are addressed in the limitations section.

## 3. Results of the Narrative Synthesis

The database searches yielded 109 records in the PHC stream and 132 records in the FSC stream. Following removal of duplicate and tangential records and relevance screening against the predefined analytical domains, 31 publications were retained as the core evidence base (Figure 1) [13,14,15,16,17,22,23,24,25,26,27,28,29,30,31,32,33,34,35,36,37,38,39,40,41,42,43,44,45,46,47]. The selected literature comprised two complementary bodies of evidence. The PHC literature addressed emergency functions, continuity of essential services, workforce and facility preparedness, information exchange, community relationships, and coordination. The FSC literature addressed conceptual development, regional and cross-sector resource mobilization, feasibility, collaborative mechanisms, exercises, and selected applications. None of the included publications directly evaluated a complete PHC-integrated FSC model.

### 3.1. PHC Emergency Functions and Continuity Requirements

The PHC literature described primary care and PHC as contributors across preparedness, response, and recovery. Reported functions included first-contact assessment, continuity of routine and chronic care, prevention, community engagement, referral, and support for redistribution of appropriate care away from hospitals during periods of exceptional demand [22,23,24,25]. Continuity of essential services was a recurrent theme. COVID-19 studies showed that emergencies may simultaneously increase healthcare needs and disrupt routine service delivery. Nyasulu and Pandya emphasized maintenance of essential services [26], Garg et al. examined PHC facility preparedness for continued outpatient care [27], and Khatri et al. identified interruption of healthcare delivery and utilization as a recurrent consequence of public-health emergencies [28].

Emergency capability also depended on functional preparedness rather than the physical presence of facilities alone. Alrayyes et al. examined both provider competency and facility preparedness [29], while Ward et al. identified regional networks, leadership, service delivery, health information, workforce, medical products and technologies, and financing as interdependent preparedness domains [30]. Studies of family physicians and primary-care systems during COVID-19 reported substantial workforce and practice-management pressures associated with emergency response [31,44]. Specific PHC functions relevant to surge planning included risk communication with family physicians [32], pharmaceutical services [33], health information integration [34], and preparedness support for vulnerable populations through established primary-care relationships [35].

Overall, the PHC evidence indicates that emergency readiness requires both the ability to assume additional emergency functions and the ability to maintain essential routine services.

### 3.2. Subnational and Multisectoral Coordination

The literature identified variation in how national, or system-level preparedness was translated into frontline PHC activity. Midtbø et al. reported differences in municipal pandemic strategies and in the involvement of emergency PHC services [36]. Ezenwaka et al. identified stakeholder roles and multilevel and multisectoral coordination mechanisms as important components of COVID-19 response [37]. Zaidi et al. described stewardship challenges associated with integrating PHC into a fragmented health security system [38], while comparative evidence from South-East Asia demonstrated substantial variation in the organization and use of PHC during the pandemic [39].

These studies indicate that coordination between national policy and frontline implementation involves multiple existing actors and organizational levels rather than a single uniform mechanism.

### 3.3. Development and Application of Flexible Surge Capacity

The FSC literature describes an extension of conventional surge planning beyond resources contained within an individual healthcare institution. The initial FSC publication proposed identifying additional capabilities across healthcare, public services, and society [13], while regional FSC emphasized coordinated mobilization of additional resources when conventional healthcare capacity becomes insufficient [40].

Later studies examined feasibility, collaboration, and implementation. The Bangkok feasibility study identified willingness to participate together with educational and preparedness gaps [15]. The FSC conceptual framework emphasized complexity and collaboration [14], while simulation and multilevel exercises examined leadership and cross-organizational coordination [41,42,43]. Applied examples included the use of FSC in a community-based home-isolation center during COVID-19 [16], hospital evacuation preparedness [43], and consideration of nontraditional facilities as potential alternative care sites [17].

The FSC evidence base remained predominantly conceptual, feasibility-based, simulation-based, or context-specific. None of these studies established the comparative effectiveness of FSC on mortality, morbidity, hospital utilization, resilience, or recovery.

### 3.4. Areas of Convergence

Comparison of the two evidence streams identified four principal areas of convergence. First, the PHC requirement to preserve essential services corresponded to the FSC concept of preparing additional personnel, facilities, supplies, and systems before routine capacity became insufficient [13,15,26,27,28,31,44,45].

Second, PHC contributed functions that extended beyond acute treatment, including first-contact assessment, chronic-care continuity, pharmaceutical services, surveillance, communication, referral, and community linkage [22,23,24,25,32,33,34,35,46]. These functions broaden the types of capabilities that could potentially be considered within an FSC model.

Third, the regional and collaborative orientation of FSC corresponded to the multilevel and multisectoral coordination challenges identified in the PHC literature [14,36,37,38,39,40,41,47,48].

Fourth, both bodies of literature identify community-level capability as relevant to preparedness and response. The PHC literature emphasized community relationships and vulnerable populations [35,48], while FSC studies demonstrated the feasibility of selected community-based applications [15,16].

These areas of convergence formed the basis for the proposed PHC-integrated FSC framework. The synthesis also identified unresolved requirements concerning governance, workforce protection, clinical accountability, information exchange, activation, and equity; these are considered in the Discussion (Table 1).

### 3.5. Adaptations and Safeguards Required for PHC-Integrated FSC

The synthesis identified several areas in which incorporation of PHC into FSC would require additional safeguards. These concerned governance and multilevel coordination, workforce preparedness and protection, clinical accountability, information sharing, activation and deactivation, and equity. Governance-related evidence emphasized predefined leadership, stewardship, coordination, and collaborative arrangements [14,30,37,38,39,40,41,42,47,48]. Workforce studies identified provider competence, training, preparedness, and the effect of emergency demand on routine primary care as important constraints [15,29,30,31,42,44]. Evidence concerning PHC facility preparedness, pharmaceutical services, referral pathways, and alternative care settings also indicated the need for clinical and organizational safeguards when functions are redistributed [17,27,29,33,49].

The literature further showed that health system functioning may deteriorate through service interruption, workforce pressures, information limitations, and other operational constraints rather than through bed shortages alone [28,30,31,44,45,46,50]. However, the included studies did not provide validated activation thresholds for PHC-integrated FSC. Finally, studies addressing vulnerable populations and community resilience highlighted the importance of local knowledge, access, and community relationships [16,35,47,51,52]. The evidence did not establish that PHC-integrated FSC improves equity, indicating that equity should remain an explicit consideration for future implementation and evaluation.

## 4. Discussion

This review brought together two bodies of literature that have largely developed separately: evidence concerning PHC during emergencies and the conceptual and applied literature on FSC. The synthesis suggests that incorporating PHC into FSC would do more than add another source of personnel or facilities. It would require FSC planning to account simultaneously for additional emergency demand and the preservation of essential frontline healthcare. PHC-integrated FSC is therefore proposed as a continuity-and-surge model rather than solely an expansion of acute treatment capacity.

### 4.1. From Additional Capacity to Continuity-and-Surge

Conventional surge planning has commonly focused on the capacity of hospitals and emergency services to absorb increasing numbers of patients [53]. The PHC literature demonstrates that emergencies also create requirements for first-contact care, chronic-disease management, medication continuity, surveillance, vaccination, communication, referral, and maintenance of routine services [22,23,24,25,26,27,28,32,33,34,35,45,46,54]. This distinction changes how reserve capacity should be interpreted. Studies during COVID-19 demonstrated substantial pressures on family physicians and primary-care practices while essential services still needed to be maintained [26,27,28,31,44]. A functioning PHC clinic or workforce therefore cannot automatically be considered available surge capacity merely because it exists. Its baseline workload and the consequences of redeployment must also be considered.

PHC-integrated FSC should consequently be based on functional reserve rather than nominal numbers of staff, facilities, or beds. Additional capability should supplement rather than deplete essential PHC services. Mobilizing PHC resources without considering baseline demand may transfer a capacity problem from one part of the healthcare system to another. At the same time, PHC broadens what may constitute useful surge capability. Depending on the hazard and local system, this could include community-based assessment, medication continuity, vaccination or testing, home and outreach services, information exchange, and referral coordination, rather than only additional acute-care beds [13,25,33,34,40,45,54].

### 4.2. The Subnational Level as an Operational Coordination Interface

The term “missing middle” should not be interpreted as implying that national, regional, municipal, or local emergency structures do not exist. Rather, the reviewed evidence and current preparedness frameworks point to an operational challenge in translating national preparedness into coordinated frontline delivery [47,55,56]. Variation in municipal PHC involvement, multilevel coordination mechanisms, stewardship, and pandemic responses across health systems illustrates this problem [36,37,38,39,47]. Regional FSC was similarly developed around the mobilization of capabilities across organizational boundaries when conventional resources become insufficient [40].

The proposed role of the subnational level is therefore to provide an operational interface through which existing hospitals, PHC, public health, emergency medical services, emergency management, private providers, pharmacies, and community organizations can match emerging needs with available capabilities. This does not require a new administrative authority; it requires predefined relationships, decision rights, communication channels, and accountability between existing actors [47,55,56,57].

The proposed PHC-integrated FSC model should be interpreted as complementary to, rather than duplicative of, existing health-emergency coordination frameworks. The WHO National Health Emergency Alert and Response Framework addresses functions including detection, notification, risk assessment, activation, coordination, response, and operational review across national, subnational, and local levels. The specific contribution proposed here is narrower and operational: to provide a conceptual basis for linking PHC functions and continuity requirements with pre-identified and prepared additional capabilities when routine functional capacity becomes insufficient. Table 2 summarizes this distinction.

The comparison shows that PHC-integrated FSC is not intended to establish a parallel preparedness structure or an additional administrative level. Its proposed role is to operate within existing emergency governance arrangements by making PHC continuity, functional reserve, graded activation, and the governed mobilization of additional cross-sector capabilities more explicit at the operational interface between system-level preparedness and frontline delivery.

### 4.3. Prepared Flexibility Rather than Improvisation

A consistent finding from the FSC literature is that potential resources are not equivalent to usable surge capacity. The Bangkok feasibility study identified willingness to participate alongside educational gaps [15], while FSC exercises highlighted leadership, training, collaboration, and organizational preparedness [41,42,43,57]. This principle becomes more important when PHC, community organizations, alternative facilities, or non-health personnel are involved. Clinics, pharmacies, hotels, municipal facilities, volunteers, or other community resources become operational capabilities only when their roles, competence, supervision, logistics, communication, and accountability have been specified in advance [15,16,17,43].

Non-health personnel may contribute selected supportive functions, such as first aid, transport, logistics, communication, or welfare support, when these tasks are predefined and appropriate to local arrangements. Such participation should be based on task-specific training, demonstrated competence, supervision, occupational protection, and clear escalation pathways; non-health personnel should not substitute for health professionals outside defined competencies [15,51,52,54]. FSC should therefore represent pre-identified and prepared flexibility, not ad hoc improvisation after routine systems have failed.

### 4.4. Governance, Workforce, and Clinical Safety

PHC-integrated FSC requires explicit governance. Multilevel coordination, stewardship, leadership, financing, and information exchange are recurrent requirements in both the PHC and FSC evidence and in broader preparedness guidance [14,30,37,38,39,40,41,47,55,56,57]. Preparedness arrangements should therefore identify activation authority, role allocation, resource ownership, financing, information-sharing responsibilities, and deactivation procedures before an emergency occurs. Workforce arrangements require similar preparation. Provider competency, training, supervision, occupational protection, and the effect of redeployment on routine services are important constraints on PHC emergency participation [15,29,30,31,42,44,47]. Credentialing and scope-of-practice arrangements will depend on the workforce and jurisdiction but should be resolved prospectively rather than during activation.

Clinical flexibility also requires boundaries. Evidence on PHC facility preparedness, pharmacy functions, referral needs, and alternative care locations indicates that shifted care must remain clinically governed [17,27,29,33,49]. Locally developed arrangements should specify which patients and services can safely be managed in PHC or alternative settings; eligibility and exclusion criteria; acuity limits; reassessment intervals; recognition of deterioration; escalation and transfer pathways; prescribing and medication storage; diagnostic access; documentation and information access; consent and safeguarding; infection prevention; and named senior clinical accountability [16,17,29,33,49,56].

The precise configuration will vary by hazard. Infectious-disease outbreaks may emphasize surveillance, infection prevention, isolation, testing, and vaccination; mass-casualty trauma may require rapid triage, stabilization, and transfer; cyber disruption may impair records, communication, diagnostics, and referral coordination; chemical events may require decontamination, exposure-specific protection, and specialized referral; conflict may add security, access, displacement, supply, and workforce-protection constraints; climate-related events may disrupt infrastructure and transport; prolonged emergencies may increase chronic-care and medication needs [30,54,55,56]. FSC may provide a general organizational architecture, but the functions activated must remain hazard-, context-, and capability-specific.

### 4.5. Activation Based on Functional Capacity

The reviewed literature does not provide a validated threshold for activating PHC-integrated FSC. Nevertheless, it indicates that healthcare function may deteriorate for reasons other than lack of beds, including workforce shortages, interruption of essential services, information-system problems, supply constraints, loss of infrastructure, and referral bottlenecks [28,30,31,44,45,46,50,55]. Activation should therefore be linked to a mismatch between demand and functional capacity, rather than hospital occupancy alone. Potential indicators may include workforce absence, interruption of essential PHC services, referral congestion, ambulance delays, diagnostic or supply-chain limitations, infrastructure failure, and projected demand. These indicators are proposed from the synthesis and should not be interpreted as validated thresholds.

A graded approach is therefore proposed: *readiness → partial activation → broader/full activation → stand-down*. Readiness would include resource mapping, agreements, training, and situational awareness; partial activation would mobilize selected capabilities in response to emerging gaps; broader activation would occur where multiple components of routine capacity become insufficient; and stand-down would restore routine services and incorporate structured learning. This model requires empirical validation.

### 4.6. Community Partnership and Equity

Both evidence streams support a role for community-level capabilities, but communities should not be conceptualized simply as an informal reserve workforce. Primary-care providers may possess detailed knowledge of local health needs and vulnerable populations [35], and community-driven approaches have been associated with PHC resilience during shocks [48]. PHC-integrated FSC should therefore involve communities and civil-society organizations in preparedness, planning, communication, and evaluation rather than only at the point when formal services are overwhelmed [55,57]. Where community members undertake operational roles, task-specific training, protection, supervision, and accountability remain necessary [15,51,52].

Equity should similarly be incorporated prospectively. Alternative modes of service delivery may benefit some groups while increasing barriers for others, particularly populations affected by geography, disability, language, digital exclusion, socioeconomic disadvantage, transport limitations, or dependence on regular medicines and complex care [35,48]. The reviewed evidence does not demonstrate that PHC-integrated FSC will automatically improve equity. Equity should therefore be treated as both a design requirement and an outcome for evaluation, with indicators selected and, where appropriate, co-designed with affected communities [51,52].

### 4.7. Information as a Surge Capability

Information exchange should be regarded as part of functional surge capacity rather than as a background administrative activity. PHC preparedness studies identified health information and risk communication as important operational domains [30,33,35]. PHC-integrated FSC would require timely sharing of service availability, workforce status, referral capacity, medication shortages, operational constraints, and emerging community needs. Without shared situational awareness, additional resources may exist but remain poorly matched to actual demand.

### 4.8. What FSC Adds—And What Remains Unproven

FSC overlaps with conventional surge, contingency/crisis capacity, mutual aid, regional load balancing, alternative care sites, task shifting/task sharing, and deployable emergency resources, but its proposed distinguishing feature is the advanced identification, preparation, and governed mobilization of additional capabilities across organizational and sectoral boundaries [13,14,15,16,17,40,41,42,43,53]. Task shifting may be one operational strategy within FSC, but it redistributes defined tasks among personnel, whereas FSC concerns the broader preparedness and mobilization of additional personnel, facilities, supplies, systems, logistics, and community capabilities. Table 1 summarizes these relationships (see Section 3.4).

The present synthesis extends this concept by explicitly incorporating PHC functions and continuity requirements. The proposed contribution is therefore neither a new administrative structure nor a claim that FSC is superior to existing emergency-response mechanisms. It is a framework for considering how additional capability might be mobilized without losing essential healthcare close to communities. Importantly, none of the reviewed studies evaluated the complete PHC-integrated FSC model proposed here. The downstream effects shown in the conceptual framework—including continuity of care, reduced avoidable hospital burden, resilience, and equity—should therefore be treated as hypotheses for future evaluation rather than demonstrated benefits.

### 4.9. Evidence Limitations and Research Priorities

The PHC evidence is heterogeneous in design, hazard, profession, health system, and outcome [22,24,30,48]. The FSC evidence base is considerably smaller and consists mainly of conceptual publications, feasibility studies, simulations, and context-specific applications [13,14,15,16,17,40,41]. A further limitation is that FSC originated within the present author’s research network, and a substantial proportion of the FSC literature involves the author or recurring collaborators. These publications are necessary to describe the provenance and development of the concept, but their concentration within a limited research network raises the possibility of intellectual allegiance. Independent replication and comparative evaluation are therefore particularly important.

Future research should move beyond feasibility and perceived preparedness. Relevant outcomes include maintenance of essential services, time to activation, workforce effects, medication continuity, referral delays, transfer rates, adverse events, patient experience, avoidable emergency-department utilization, equity of access, costs, and time to restoration of routine services. Evaluative studies should prespecify the comparator (for example, existing regional coordination or usual surge arrangements), hypothesized process mediators such as activation time, referral performance, workforce availability, and continuity of essential services, and clinically meaningful time horizons for both emergency response and recovery.

Comparative studies should assess FSC-supported arrangements against existing approaches rather than assuming benefits from additional nominal capacity. Where feasible, studies should also report unsuccessful activations, unintended consequences, service displacement, and negative implementation experiences.

### 4.10. Implications for Policy and Practice

**Figure 2:**
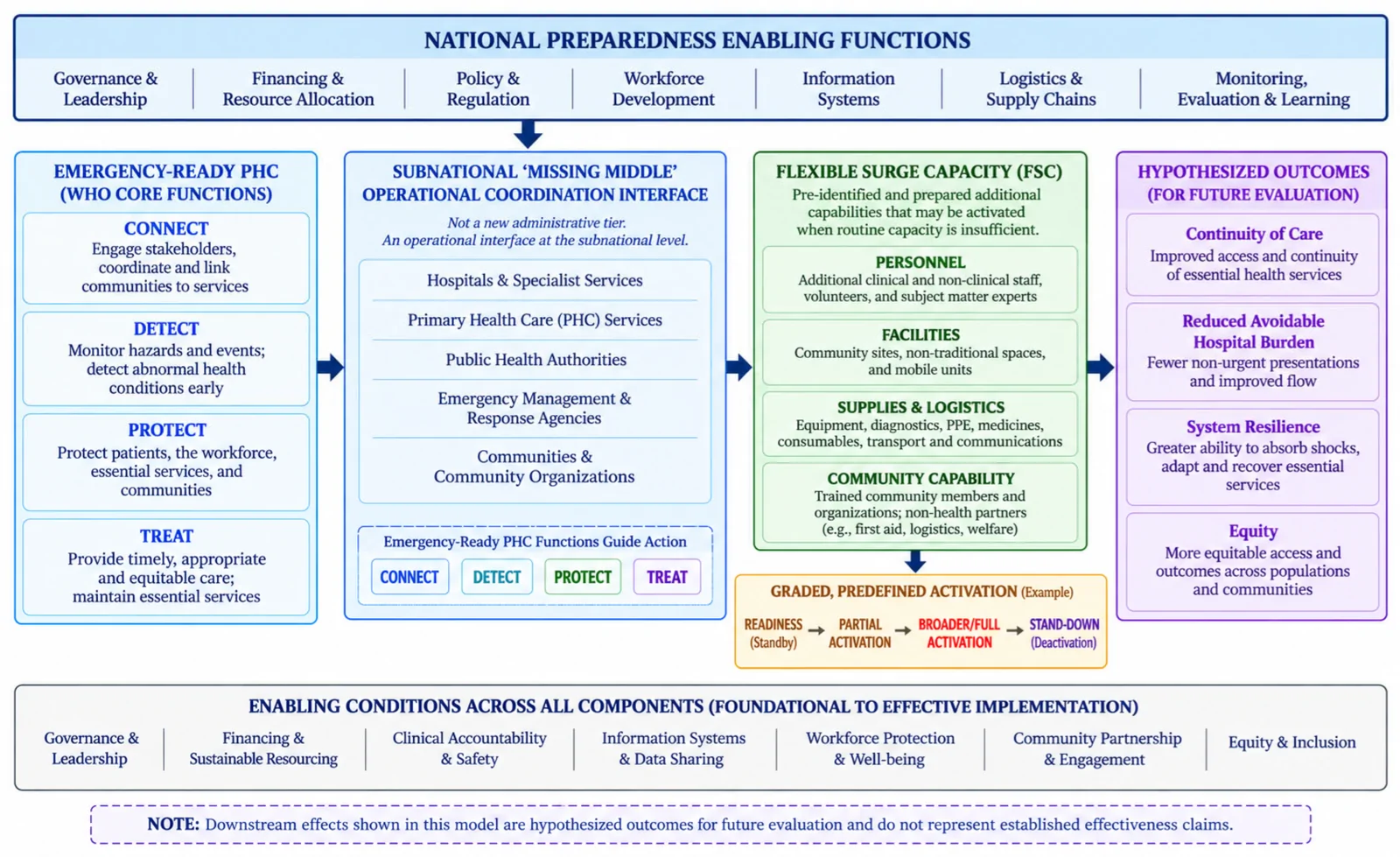
Conceptual model of the subnational “missing middle” linking national preparedness with healthcare and community capabilities through PHC-integrated flexible surge capacity. The model depicts the subnational level as an operational coordination interface rather than a new administrative tier. Connect, detect, protect, and treat represent emergency-ready PHC functions; “protect” refers to the protection of patients, the workforce, essential services, and communities. FSC represents pre-identified and prepared additional capabilities that may be activated through a proposed graded sequence from readiness to partial activation, broader/full activation, and stand-down. Governance, financing, clinical accountability, information systems, workforce protection, community partnership, and equity are enabling conditions. Activation thresholds and downstream effects are conceptual and require empirical validation; the outcomes shown are hypotheses for future evaluation rather than established effectiveness claims.

PHC-integrated FSC is not intended to replace established emergency management structures, regional coordination, mutual aid, or adequately resourced routine PHC. Its proposed contribution is to connect them more deliberately [14,37,38,39,40,41,45,47,55,56]. PHC identifies the functions that need to remain available close to communities, including continuity of essential services, first-contact care, communication, referral, and community linkage [22,23,24,25,26,27,28,32,33,34,35,45,46,54], while FSC provides an approach for identifying and preparing additional capabilities when routine capacity becomes insufficient [13,14,15,16,17,40,41,42,43]. The subnational level may therefore serve as an operational interface through which these functions and capabilities are coordinated across existing actors [36,37,38,39,40,47,55,56]. The central proposition is therefore that surge planning should address continuity and additional capacity simultaneously. Whether this approach improves continuity, reduces avoidable hospital use, strengthens resilience, improves equity, or changes patient outcomes remains an empirical question (Table 3 and Figure 2).

Preparedness should be assessed using operational indicators rather than solely by the existence of written emergency plans [14,37,38,39,50,55]. In addition to activation and deactivation criteria, evaluation should include continuity of essential PHC services, workforce protection, information-sharing performance, and clinical accountability. Equity assessment should move beyond aggregate access measures and, where data governance permits, examine differences in access, time to care, medication continuity, and referral completion across locally relevant population groups [35,48,51,52]. For Indigenous and other affected communities, indicators and governance arrangements should, where appropriate, be locally determined and co-designed. Figure 2 summarizes the proposed PHC-integrated FSC model, positioning the subnational level as an operational coordination interface between national preparedness, frontline healthcare, public health, emergency management, and community capabilities.

### 4.11. Strengths and Limitations

A strength of this review is the deliberate integration of two complementary evidence streams: the literature on the functions and requirements of primary health care during emergencies and the conceptual and applied literature on flexible surge capacity. The two streams were examined separately and then synthesized across 31 core publications to identify where PHC emergency functions correspond to FSC capabilities and where additional governance, clinical, workforce, information, equity, and activation safeguards would be required.

Several limitations should be acknowledged. First, this was a narrative conceptual review rather than a systematic review, and the literature was selected for conceptual relevance rather than exhaustive coverage. Second, the PHC literature is heterogeneous in terms of hazards, health system settings, study designs, professional groups, and outcomes, which limits direct comparison between studies. Third, the FSC evidence base remains relatively small and consists mainly of conceptual work, feasibility studies, simulation exercises, and context-specific applications; evidence of effectiveness on patient- or system-level outcomes is therefore limited. Finally, FSC originated within the present author’s research network, and a substantial proportion of the available FSC literature involves the author or recurring collaborators. Although citation of these studies is necessary to describe the provenance and development of the concept, this concentration of evidence introduces a potential risk of intellectual allegiance. To reduce this risk, citations from the originating research group were restricted to publications necessary to define, develop, or document FSC, while independent sources were preferentially used for broader claims concerning preparedness, governance, clinical safety, community participation, and health system response. This limitation further highlights the need for independent replication, comparative evaluation, and publication of unsuccessful or negative implementation experiences.

## 5. Conclusions

Primary health care is an important component of health-emergency preparedness because it supports continuity of essential services, first-contact care, surveillance, medication access, chronic disease management, communication, referral, and community linkage. Flexible surge capacity provides a complementary framework for identifying and preparing additional personnel, facilities, supplies, systems, and community capabilities when routine functional capacity becomes insufficient.

Integrating PHC into FSC at the subnational level may provide a continuity-and-surge approach in which additional emergency capability is coordinated while essential frontline services are protected. Such integration requires predefined governance, graded activation, workforce and clinical safeguards, information sharing, community partnership, and explicit attention to equity. It should complement, rather than replace, adequately resourced routine PHC and existing emergency-response structures.

The proposed model is conceptual. Its effects on continuity of care, hospital demand, resilience, equity, patient outcomes, and recovery have not been established and should be evaluated through independent, comparative, and context-sensitive research.

## Figures and Tables

**Table 1:** Comparison of flexible surge capacity with related surge and emergency response approaches.

Approach	Typical Level/Resource Base	Activation and Clinical Scope	Relationship to FSC
Conventional institutional surge	Hospital; internal staff, space, supplies, systems	Institutional incident command; expansion of usual hospital functions	FSC extends the resource base beyond a single institution.
Contingency/crisis capacity	Hospital or health system; existing healthcare resources	Activated as demand and scarcity increase; may involve modification of usual processes or standards	FSC may operate alongside these states but focuses on additional prepared resources across boundaries.
Mutual aid	Inter-organizational; resources loaned or shared by partner organizations	Pre-agreed request/authorization; scope depends on the resource shared	FSC can incorporate mutual aid but also maps nontraditional facilities, personnel, logistics, and community capabilities before activation.
Regional load balancing	Regional network of existing healthcare facilities	Regional coordination; redistribution of patients or workload across functioning providers	FSC may support load balancing but additionally prepares resources not routinely used for the same purpose.
Alternative care sites	Temporary or repurposed facilities	Activated for defined patient groups or functions with site-specific clinical governance	Alternative sites can be one FSC resource, but FSC is broader than the site itself.
Emergency medical teams	Deployable national/international teams with defined capabilities	Activated through governmental or humanitarian mechanisms; specialized clinical scope	EMTs are deployable external capacity; FSC primarily emphasizes locally/sub-nationally mapped and governed capabilities.
Task shifting/task sharing	Within or across health-workforce roles; may include appropriately trained lay/community workers	Redistribution of defined tasks under specified competency, supervision, governance, and escalation arrangements	Task shifting can be used within FSC, but it changes who performs a defined task; FSC is broader and concerns advance preparation and mobilization of additional capabilities across organizational and sectoral boundaries.
Flexible surge capacity	Subnational/cross-sector; healthcare and appropriately prepared nontraditional resources	Predefined graded activation; locally specified clinical/nonclinical scope, accountability, financing, and safeguards	Proposed distinguishing feature is advance mapping and governed mobilization across organizational and sectoral boundaries; added clinical effectiveness remains to be demonstrated.

**Table 2:** Relationship between the WHO National Health Emergency Alert and Response Framework and the proposed PHC-integrated flexible surge capacity model.

Function	WHO National Health Emergency Alert and Response Framework	PHC-Integrated FSC Contribution
National/subnational/local coordination	Explicitly covered	Uses existing coordination structures; does not create a new tier
Detection, notification, risk assessment	Explicitly covered	PHC contributes frontline detection, surveillance, communication and local information
Emergency activation and incident coordination	Explicitly covered	Adds graded activation of pre-identified PHC and cross-sector capabilities based on functional capacity
Resource coordination	Covered at system-response level	Focuses specifically on mapping and preparing additional personnel, facilities, supplies, logistics and community capabilities before activation
Continuity of essential PHC	Relevant within health system response	Makes protection of routine PHC an explicit constraint on surge activation
Clinical redistribution outside hospitals	Not the primary focus of the framework	Specifies safeguards for shifting appropriate functions to PHC or alternative settings
Community and nontraditional resources	Multisectoral participation is recognized	Specifies preparedness, competency, supervision and governance requirements for their operational use
Evaluation	Performance and operational review included	Proposes PHC-specific continuity, workforce, equity and functional-capacity indicators

**Table 3:** The table summarizes implementation domains for PHC-integrated FSC and translates the conceptual synthesis into practical preparedness actions and illustrative indicators.

Implementation Domain	Required Arrangements	Illustrative Indicators
Governance and connection	Formal PHC representation; public health/emergency-management links; mutual aid; community participation	Named activation authority; PHC/community participation in plans and exercises
Capability mapping	Workforce skills; facilities; pharmacies; equipment; transport; communications; population needs	Capability registers current; alternative sites assessed; backup communications tested
Continuity and clinical roles	Medication access; chronic care; vaccination; triage; surveillance; referral; home/outreach care	Minimum service levels defined; continuity plans exercised
FSC activation safeguards	Credentialing; supervision; indemnity; infection prevention; data access; logistics; activation/deactivation	Graded activation/deactivation criteria defined; activation time; trained staff; agreements in place; routine-service depletion monitored; corrective actions completed
Community partnership and equity	Co-design; compensated participation; accessible communication; locally legitimate governance; data safeguards	Participation breadth; communication reach; access, time-to-care, medication continuity, and referral outcomes stratified by locally relevant geography and population groups
Learning and accountability	Exercises; after-action review; public reporting; funded corrective plans	Exercise frequency; corrective actions closed; continuity and equity outcomes reported

## Data Availability

No new data were created or analyzed in this study. Data sharing is not applicable to this article.

## Supplementary Materials

The following supporting information can be downloaded at https://www.mdpi.com/article/10.3390/healthcare14183074/s1: Table S1: Search string and databases; Table S2: The characteristics of the included studies in this review.
