# Supplementary material for: Integrating Primary Health Care into Flexible Surge Capacity: Bridging the Subnational “Missing Middle” in Health Emergency Preparedness
Source: Healthcare (Basel). 2026 Sep 18;14(18):3074. doi: 10.3390/healthcare14183074 (PMC13606306; doi:10.3390/healthcare14183074)
Supplement: Supplementary file 1 [file healthcare-14-03074-s001.zip › healthcare-4531098-supplementary.pdf]

Supplementary Tables

Table S1: Search string and databases: The same final PHC search expression was used in PubMed, Scopus, and Web of Science: “primary health care” AND “primary care” AND “health emergency preparedness.” The FSC stream used the exact phrase “flexible surge capacity” across the same three databases. Where no database-specific field restriction was applied, this is stated explicitly rather than retrospectively constructing database-specific syntax. The exact search syntax, search fields/interface, limits, and search dates are provided in this Supplementary Table.

Evidence stream	Database	Exact search syntax	Field/interface	Limits	Search date
**PHC/emergency preparedness**	PubMed	"Primary health care" AND "primary care" AND "health emergency preparedness"	Default search interface; no additional field restriction	English; 2020–July 2026	15–25 July 2026
**PHC/emergency preparedness**	Scopus	same search syntax	Default search interface; no additional field restriction	English; 2020–July 2026	15–25 July 2026
**PHC/emergency preparedness**	Web of Science	same search syntax	Default search interface; no additional field restriction	English; 2020–July 2026	15–25 July 2026
**FSC**	PubMed, Scopus, Web of Science	"flexible surge capacity"	Default search interface; no additional field restriction	English; 2020–July 2026*	15–25 July 2026

Table S2: The characteristics of the included studies in this review.

Author, date and journal	Main findings	Comment
1. Lamberti-Castronuovo A, Valente M, Barone-Adesi F, Hubloue I, Ragazzoni L. 2022. International Journal of Disaster Risk Reduction. Primary health care disaster preparedness: A review of the literature and the proposal of a new framework.	Reviews disaster preparedness in PHC and proposes a framework for organizing PHC preparedness.	Core PHC paper. Include. Directly establishes PHC preparedness functions and requirements that can subsequently be mapped onto FSC.
2. Alesi A, Bortolin M, Ragazzoni L, Lamberti-Castronuovo A. 2023. Prehospital and Disaster Medicine. Primary Health Care and Disasters: Applying a "Whole-of-Health System" Approach through Reverse Triage in Mass-Casualty Management.	Positions PHC within a whole-health-system disaster response and discusses redistribution of patients/functions through reverse triage.	Core PHC–surge bridge. Include. Particularly relevant to moving appropriate care away from hospitals while preserving system function.
3. Makin S, Ross D. 2022. BMJ Military Health. Priorities of primary care in disaster medicine.	Argues that primary care's role in disasters has been poorly defined despite its adaptability, broad clinical scope, and proximity to communities.	Include. Strong justification for deliberately incorporating primary care/PHC into FSC rather than treating surge as hospital-centred.
4. Desborough J, Dykgraaf SH, et al. 2021. Family Practice. Lessons for the global primary care response to COVID-19: a rapid review of evidence from past epidemics.	Finds that primary-care and public-health providers have important responsibilities during health emergencies and synthesizes lessons from previous epidemics relevant to COVID-19.	Include. Provides broad evidence on PHC emergency functions and preparedness requirements.
5. Dykgraaf SH, Parkinson A, et al. 2025. BMC Primary Care. Ten pressure points in primary care during COVID-19: findings from an international narrative review.	Describes the major pressures placed on primary-care practitioners and services during COVID-19 while they contributed to the health-system response.	Highly relevant. Supports the argument that PHC cannot simply be regarded as unused reserve capacity; FSC must protect routine PHC as well as generate surge.
6. Khatri RB, Endalamaw A, et al. 2023. Archives of Public Health. Preparedness, impacts, and responses of public health emergencies towards health security: qualitative synthesis of evidence.	Shows that emergencies disrupt service delivery and utilisation while simultaneously increasing health-service needs, with impacts varying by context.	Include. Supports continuity-of-care requirements and the need for adaptable, context-sensitive surge arrangements.
7. Theron E, Smith WP. 2026. African Journal of Primary Health Care & Family Medicine. Emergency preparedness and disaster management for primary health care in Africa from the perspective of climate hazards and extreme weather events.	Examines climate hazards as threats to community health and healthcare provision, particularly where underlying vulnerability reduces system capacity.	Include. Adds hazard-specific PHC preparedness and demonstrates why FSC configurations cannot be entirely hazard-neutral.
8. Ward A, Martin S, et al. 2024. Nursing Outlook. Enhancing primary healthcare nurses' preparedness for	Identifies regional networks, clinical leadership, service delivery, information, workforce, medical	Excellent fit. Almost directly matches the enabling conditions used in the PHC-integrated FSC framework.

climate-induced extreme weather events.	products/technology and financing as important preparedness domains.	
9. Calma KR, Halcomb E, Watson KE, Mathew S, Ranse J. 2026. Australian Journal of Primary Health. The role of primary healthcare nurses in disasters due to natural hazards: a scoping review.	Maps PHC nursing roles across prevention, preparedness, response and recovery.	Include. Broadens FSC workforce thinking beyond emergency/hospital personnel and supports PHC participation across the disaster cycle.
10. Alrayyes MA, Alaila BH, et al. 2023. Prehospital and Disaster Medicine. The Emergency Preparedness of Level-Four Primary Health Care Centers in the Gaza Strip: An Assessment of Primary Care Providers' Competencies and Facilities Preparedness.	Examines PHC-provider competencies and facility preparedness in a setting where recurrent emergencies have overwhelmed hospitals and increased the need for PHC participation.	Include. Useful empirical evidence concerning whether PHC facilities and personnel are actually capable of accepting emergency functions.
11. Garg S, Basu S, Rustagi R, Borle A. 2020. JMIR Public Health and Surveillance. Primary health care facility preparedness for outpatient service provision during the COVID-19 Pandemic in India.	Assesses preparedness of PHC facilities to continue outpatient services during the pandemic.	Include. Directly supports continuity of essential services as a condition of PHC-integrated FSC.
12. Nyasulu J, Pandya H. 2020. African Journal of Primary Health Care & Family Medicine. The effects of coronavirus disease 2019 pandemic on the South African health system: A call to maintain essential health services.	Highlights disruption of essential health services during COVID-19 and argues for maintaining routine care during emergency response.	Include. Important for the manuscript's argument that surge activity must not cannibalize essential PHC services.
13. Santos-Pinto CB, Osorio-De-Castro CS, et al. 2021. Pharmacy Practice. The role of primary care pharmacists in the response to the Zika epidemic.	Examines primary-care pharmacy participation during the Zika public-health emergency.	Include. Supports medication access/pharmaceutical continuity and inclusion of pharmacies in the FSC resource map.
14. Kain NA, Jardine CG. 2020. Canadian Family Physician. "Keep it short and sweet": Improving risk communication to family physicians during public health crises.	Addresses risk communication to family physicians during public-health crises.	Include. Provides specific support for the information/communication component of PHC preparedness.
15. Midtbø V, Johansen IH, Hunskaar S. 2023. BMC Health Services Research. The association between municipal pandemic response and COVID-19 contacts to emergency primary health care services: an observational study.	Shows that municipalities used different pandemic strategies and involved emergency PHC services to varying degrees.	Very important for the "missing middle." Demonstrates how subnational/municipal decisions affect frontline PHC activity.
16. Ezenwaka U, Mbachu C, Onwujekwe O. 2025. Health Research Policy and Systems. A scoping review of the roles of stakeholders and coordination mechanisms for enhanced multi-	Reviews stakeholder roles and mechanisms for multisectoral and multilevel coordination during COVID-19.	Include. Direct support for the subnational coordination interface and cross-sector governance required for FSC.

sectoral and multi-level interventions in COVID-19 response in Nigeria.		
17. Houghton N, Bascolo E, Zavaleta C, et al. 2025. Lancet Regional Health – Americas. Community-driven strategies for primary health care resilience in response to shocks in Latin America and the Caribbean: a scoping review and expert consultation.	Examines community-driven strategies for strengthening PHC resilience during shocks.	Include. Supports community partnership as a capability and governance relationship rather than simply a pool of spare resources.
18. Zaidi S, Zaidi R, Hussain S, Safi MM. 2025. BMJ Global Health. Stewarding COVID-19 health systems response in Pakistan: what more can be done for a primary health care approach to future pandemics?	Applies a PHC perspective to Pakistan's COVID-19 response and derives stewardship lessons for better integration of PHC in future pandemics.	Include. Strong governance/subnational-integration paper, especially relevant to fragmented systems.
19. Edelman A, Marten R, Dhillon I, et al. 2025. BMJ Global Health. Primary health care in the context of the COVID-19 pandemic in 10 South-East Asian countries: a cross-case synthesis with lessons for future health systems strengthening.	Cross-country synthesis of PHC experience during COVID-19, drawing lessons for future system strengthening.	Include. Provides comparative evidence that PHC emergency roles and constraints vary across health-system contexts.
20. Mathews M, Ryan D, Hedden L, et al. 2025. Human Resources for Health. Surge capacity and practice management challenges of Canadian family physicians during COVID-19: a qualitative study.	Examines surge-capacity and practice-management challenges experienced by family physicians during COVID-19.	Core bridge paper. Directly links primary care to surge capacity and supports protecting baseline workforce and practice functions.
21. Simsek P, Kako M, et al. 2024. Progress in Disaster Science. Scoping review of exploring the roles of primary care providers to increase disaster preparedness of vulnerable populations.	Finds primary-care providers well positioned to support disaster preparedness because of their proximity to patients and knowledge of individual and community needs, while noting lack of role clarity.	Include. Particularly relevant to equity, vulnerable populations and community-facing PHC roles.
22. Tran BX, Vu HN, Duong DB, et al. 2025. Journal of Global Health. Enhancing human and animal health data integration and informed actions for pandemic preparedness at the primary healthcare level: a multisectoral conceptual framework.	Identifies challenges in integrating health information for preparedness at PHC level and proposes a multisectoral framework.	Include. Supports the information/surveillance component and cross-sector data arrangements needed in FSC.
23. Khorram-Manesh A. 2020. Health Promotion Perspectives. Flexible surge capacity—Public health, public education, and disaster management.	Introduces/develops FSC as an extension of conventional surge thinking, involving resources beyond ordinary institutional healthcare capacity.	Essential. Foundational FSC paper. Necessary to define the concept being extended to PHC.
24. Glantz V, Phattharapornjaroen P, Carlström E, Khorram-Manesh A. 2020. Sustainability. Regional Flexible Surge Capacity—A Flexible Response System.	Describes regional FSC and the need to mobilize additional resources when conventional healthcare resources are insufficient.	Essential. Establishes the regional/subnational architecture onto which PHC is being integrated.

25. Phattharapornjaroen P, Glantz V, Carlström E, et al. 2021. International Journal of Environmental Research and Public Health. The feasibility of implementing the flexible surge capacity concept in Bangkok: Willing participants and educational gaps.	Reports feasibility of FSC implementation, including willingness to participate together with educational/preparedness gaps.	Essential empirical FSC paper. Supports feasibility while also demonstrating that resources cannot simply be activated without preparation and training.
26. Phattharapornjaroen P, Carlström E, Khorram-Manesh A. 2022. Public Health. Developing a conceptual framework for flexible surge capacity based on complexity and collaborative theoretical frameworks.	Develops FSC theoretically using complexity and collaboration perspectives.	Essential conceptual paper. Provides theoretical basis for cross-organizational coordination and collaboration in PHC-integrated FSC.
27. Phattharapornjaroen P, Carlström E, Sivarak O, et al. 2022. Public Health. Community-based response to the COVID-19 pandemic: case study of a home isolation centre using flexible surge capacity.	Demonstrates a community-based FSC application through a home-isolation centre during COVID-19.	Perhaps the strongest practical bridge to the present paper. Shows FSC operating beyond conventional hospital space and interacting with community-level care.
28. Phattharapornjaroen P, Glantz V, Carlström E, Holmqvist LD, Khorram-Manesh A. 2020. Sustainability. Alternative leadership in flexible surge capacity—The perceived impact of tabletop simulation exercises on Thai emergency physicians' capability to manage a major incident.	Examines FSC-related leadership and the perceived effects of tabletop exercises on major-incident management capability.	Include. Supports the training, leadership and preparedness requirements that PHC actors would also need before incorporation into FSC.
29. Phattharapornjaroen P, Carlström E, Holmqvist LD, Sittichanbuncha Y, Khorram-Manesh A. 2023. International Journal of Disaster Risk Science. Assessing Thai Hospitals' Evacuation Preparedness Using the Flexible Surge Capacity Concept and Its Collaborative Tool.	Applies the FSC concept and collaborative tool to assess hospital evacuation preparedness.	Include. Provides operational evidence for FSC assessment/collaboration tools, although focused on hospitals rather than PHC.
30. Phattharapornjaroen P, Carlström E, Atiksawedparit P, et al. 2023. BMC Health Services Research. The impact of the three-level collaboration exercise on collaboration and leadership during scenario-based hospital evacuation exercises using flexible surge capacity concept.	Examines collaboration and leadership across three levels during FSC-based evacuation exercises.	Include. Particularly relevant to the paper's subnational interface and multilevel coordination argument.
31. Khorram-Manesh R, Khorram-Manesh A. 2024. AIMS Public Health. Can hotels be used as alternative care sites in disasters and public health emergencies—A narrative review.	Reviews hotels as possible alternative care locations during disasters and public-health emergencies.	Include as a boundary/safeguard paper. Illustrates how nontraditional facilities may expand capacity but require advance evaluation, governance and clinical safeguards.
